# The PRY/SPRY domain of pyrin/TRIM20 interacts with β_2_-microglobulin to promote inflammasome formation

**DOI:** 10.1038/s41598-021-03073-6

**Published:** 2021-12-08

**Authors:** Sei Samukawa, Ryusuke Yoshimi, Yohei Kirino, Hideaki Nakajima

**Affiliations:** Department of Stem Cell and Immune Regulation, Yokohama City University Graduate School of Medicine, 3-9 Fukuura, Kanazawa-ku, Yokohama, 236-0004 Japan

**Keywords:** Inflammation, Rheumatology, Inflammasome

## Abstract

Pyrin/TRIM20 is expressed in the neutrophils and monocytes/macrophages and regulates caspase-1 activation and interleukin-1β maturation. Although the mutations in the PRY/SPRY domain of pyrin cause familial Mediterranean fever (FMF), the mechanism of how mutated pyrin provokes excessive inflammation in FMF patients is not well understood. The present study investigated the role of pyrin/TRIM20 in inflammation and the pathogenesis of FMF. β_2_-Microglobulin (β2MG) was identified as the novel pyrin ligand binding to the PRY/SPRY domain by yeast two-hybrid screenings and co-immunoprecipitation analysis. β2MG was co-localized with pyrin not only in the HEK293 cells overexpressing these proteins but also in the monosodium urate-stimulated human neutrophils in the speck-like structures. The pyrin–β2MG interaction triggered the binding of pyrin and proline–serine–threonine phosphatase interacting protein 1 (PSTPIP1) and then the subsequent recruitment of apoptosis-associated speck-like protein containing caspase recruitment domain (ASC). Caspase-1 p20 subunit, produced by pyrin inflammasome, also interacted with the pyrin PRY/SPRY domain and inhibited the pyrin–β2MG interaction. FMF-associated pyrin mutation M694V did not affect pyrin–β2MG interaction but weakened this inhibition. Our findings suggest that β2MG functions as the pyrin ligand inducing pyrin inflammasome formation and that the FMF-associated pyrin mutations weakened negative feedback of caspase-1 p20 subunit.

## Introduction

Familial Mediterranean fever (FMF) is one of the most prevalent hereditary autoinflammatory diseases, characterized by recurrent febrile attacks with serosal inflammation^1^. The onset of FMF is usually in childhood and the patients show periodical episodes of fever with chest and/or abdominal pain, which resolve within 48–72 h. The gene responsible for FMF was identified and named *MEFV*, which is located on chromosome 16, in 1997^2,3^.

The *MEFV* gene encodes pyrin protein, which is also known as tripartite motif-containing (TRIM) 20 or marenostrin. Pyrin is a 781-amino acid and ~ 86 kDa protein, which is mainly expressed in the cytoplasm of the cells of the innate immune system, including neutrophils, eosinophils, monocytes, macrophages, and dendritic cells^4^. Pyrin belongs to the TRIM family, which numbers over 70 proteins in humans with homologs identified in many species ranging from primates to nematodes, exhibiting a wide range of activities, including the regulation of innate and adaptive immune responses^5–9^. Pyrin is comprised of several distinct domains—pyrin, B-box and coiled-coil domain in the N-terminal region followed by a PRY/SPRY domain in the C-terminus^10^.

Pyrin interacts with an adapter protein called apoptosis-associated speck-like protein with a caspase-recruitment domain (ASC) through the interaction of their respective N-terminal pyrin domains^11^. ASC oligomerizes and mediates the proteolytic activation of caspase-1, leading to proteolytic maturation and secretion of interleukin-1β (IL-1β) in cytosolic multiprotein oligomers denoted inflammasomes^12^. Therefore, it is presumed that pyrin has a proinflammatory role by forming “pyrin inflammasome” with ASC and caspase-1^13^.

So far, several pathways have been suggested to induce the pyrin inflammasome formation. One of them is proline–serine–threonine phosphatase-interacting protein 1 (PSTPIP1)-mediated pathway. PSTPIP1 binds to the B-box domain of pyrin^14^, and its mutation causes another hereditary autoinflammatory disease, pyogenic arthritis, pyoderma gangrenosum and acne (PAPA) syndrome^15^. PAPA syndrome-associated PSTPIP1 mutation, A230T, heightens its affinity for pyrin and consequently promotes the pyrin inflammasome formation^14^. However, it remains unknown what triggers the interaction between wild-type PSTPIP1 and pyrin in FMF pathology.

Because FMF-associated *MEFV* mutations are concentrated on exon 10^16^, corresponding to PRY/SPRY domain of pyrin (e.g*.* M680I, M694I/V, and V726A), this domain has been considered essential for the molecular mechanisms causing FMF. The crystal structure of the pyrin PRY/SPRY domain forms a shallow cavity, which seems to be a protein binding site, and FMF-associated mutation sites are observed around it^17^, suggesting that FMF-associated pyrin mutations affect the binding affinity of its ligands. To date, the p20 subunit of caspase-1^18^ and apoptosis-associated protein Siva-2^19^ were reported to bind to the pyrin PRY/SPRY domain.

There are two conflicting studies investigating the effect of FMF-associated pyrin mutation on the binding affinity of PRY/SPRY domain to p20. A study group reported that pyrin–p20 binding was reduced by FMF-associated mutations in PRY/SPRY domain^18^. They also showed that FMF-associated mutations and deletion of the PRY/SPRY domain reduce the effect of pyrin on the attenuation of IL-1β production. On the other hand, the other group reported that pyrin M694V mutation, the most frequent mutation found in FMF patients, showed no impacts on the interaction of pyrin with p20^20^. The result suggests that the FMF-associated mutations do not affect the pyrin–p20 interaction directly but indirectly by some other pyrin-binding proteins whose affinity for pyrin is affected by the mutations.

Although the previous studies shed some light on pyrin and FMF pathogenesis as just described, it is still unclear how the FMF-associated pyrin mutations lead to inflammasome activation and IL-1β overproduction. In the present work, we demonstrate that β_2_-microglobulin (β2MG) associates and colocalizes with pyrin in the cytoplasm. β2MG interacts with PRY/SPRY domain of pyrin, in more detail, with the hotspot of FMF-associated mutations, and promotes PSTPIP1-mediated ASC recruitment. The β2MG–pyrin association is suppressed by the p20 subunit of caspase-1. Pyrin M694V mutation promotes the β2MG–pyrin association in the presence of p20. The mutation itself does not affect pyrin–PSTPIP1 interaction in the presence of β2MG. Taken together, our results point to the importance of β2MG–pyrin interaction in the molecular mechanism of pyrin inflammasome activation in FMF.

## Results

### Yeast two-hybrid screening for pyrin-binding protein

**Figure 1:**
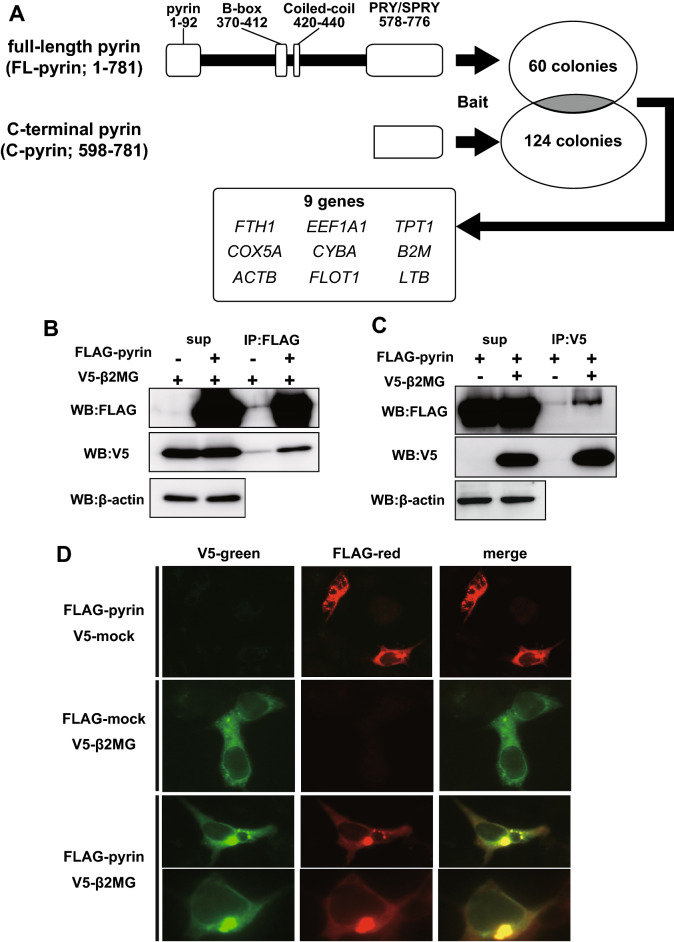
Pyrin interacts with β2MG in mammalian cells. (**A**) The yeast AH109 was transformed with Full-length pyrin (FL-pyrin) or C-terminal pyrin (C-pyrin) inserted in pGBKT7 vector as baits. As prey, human leukocyte cDNAs were inserted in pACT2 and transfected to yeast Y187 strain. After mating, FL-pyrin and C-pyrin baits yielded 60 and 124 positive colonies, respectively. Nine common genes were identified from both FL- and C-pyrin baits. (**B**,**C**) Lenti-X 293T cells expressing FLAG-tagged pyrin (FLAG-pyrin) and V5-tagged β2MG (V5-β2MG) were lysed and immunoprecipitated by FLAG Ab-conjugated agarose beads or V5 Ab-conjugated magnet beads, and then subjected to immunoblot analysis. V5-β2MG is co-immunoprecipitated with FLAG-pyrin (**A**) and vice versa (**B**). (**D**) HEK293 cells expressing FLAG-pyrin (red) and V5-β2MG (green) were subjected to immunofluorescence staining. Pyrin and β2MG shows co-localization in the cytoplasm.

Although certain bacterial toxins induce pyrin inflammasome formation^21^, FMF attacks are not always triggered by infection but some other factor including stress and menstrual cycle. Therefore, we hypothesized that some endogenous proteins expressed in neutrophil may interact with pyrin and play an important role as a damage-associated molecular pattern. To identify a molecule that interacts with pyrin, we performed a yeast two-hybrid screening assay using human leukocyte cDNA library as prey. Our use of full-length pyrin (FL-pyrin; aa 1–781) as bait yielded 60 positive colonies (Fig. 1A). To exclude false positives as much as possible before subsequent confirmatory binding assays, and to identify pyrin-binding proteins which interact with pyrin via its PRY/SPRY (B30.2) domain where mutations are often observed in patients with FMF, we also performed the screening using the C-terminal region of pyrin molecule (C-pyrin; aa 598–781) as bait. We found 124 positive colonies among which only 9 were isolated from both screenings using FL-pyrin bait and that using C-pyrin bait in common (Fig. 1A). The colony-direct PCR and sequence revealed the 9 candidates for genes encoding pyrin-binding molecules, including *FTH1*, *EEF1A1*, *TPT1*, *COX5A*, *CYBA*, *B2M*, *ACTB*, *FLOT1*, and *LTB* (Table 1).

**Table 1:** The genes obtained commonly from both yeast two-hybrid library screenings using FL- and C-pyrin baits.

Symbol/gene ID	Full name	Summary
*FTH1*/2945	Ferritin, heavy polypeptide 1	Iron storage protein
*EEF1A1*/1915	Eukaryotic translation elongation factor 1 alpha 1	Enzymatic delivery of aminoacyl tRNAs to the ribosome
*TPT1*/7178	Tumor protein, translationally-controlled 1	Regulator of cellular growth and proliferation
*COX5A*/9377	Cytochrome c oxidase subunit Va	Terminal enzyme of the mitochondrial respiratory chain
*CYBA*/1535	Cytochrome b-245, alpha polypeptide	Primary component of the microbicidal oxidase system of phagocytes
*B2M*/567	Beta-2 microglobulin	Component of human major histocompatibility class I
*ACTB*/60	Actin beta	Cytoskeleton
*FLOT1*/10211	Flotillin 1	Vesicle trafficking and cell morphology
*LTB*/4050	Lymphotoxin beta	Inducer of the inflammatory response system

### β2MG interacts with pyrin

β2MG protein is a component of major histocompatibility complex (MHC) class I molecules, which are present on the plasma membrane of all nucleated cells. In neutrophils, β2MG molecules are expressed not only on the cell surface as an MHC class I component but also inside the cells^22^. Two-thirds of β2MG expression amount in neutrophils is localized in gelatinase granules or specific granules although the physiological role of this intragranular β2MG is still unknown^22^. As we identified the *B2M* gene, which encodes β2MG protein, as the candidate for genes encoding pyrin-binding molecules by the yeast two-hybrid screening, we focused on β2MG and tried to confirm the interaction with pyrin by immunoprecipitation assay. We subcloned the full-length *B2M* cDNA into pcDNA3.1(–) vectors with V5 tag in the N-terminus and co-transfected them with FLAG-tagged pyrin expression vector into Lenti-X 293T cells. The co-immunoprecipitation analysis using anti-FLAG antibody (Ab) revealed that V5-tagged β2MG was co-precipitated with FLAG-tagged pyrin (Fig. 1B). Additionally, the reciprocal immunoprecipitation assay using anti-V5 Ab showed that FLAG-pyrin was co-precipitated with V5-β2MG (Fig. 1C). Although the other eight candidates were also subjected to co-immunoprecipitation analyses, they did not show any interaction with pyrin (data not shown).

To further confirm the pyrin–β2MG interaction, the expression vectors for FLAG-pyrin and V5-β2MG were co-transfected into human embryonic kidney (HEK) 293 cells and the localization of these fusion proteins was examined by immunofluorescence staining. As shown in Fig. 1D, the overexpressed V5-β2MG distributed homogeneously in the cytoplasm and did not show any specific structure when FLAG-pyrin was not overexpressed. On the other hand, V5-β2MG co-localized with pyrin in the speck-like structures in the perinuclear position in more than 90% of the cells in which both FLAG-pyrin and V5-β2MG were overexpressed.

### β2MG binds to the hotspot of FMF-associated mutations of pyrin

**Figure 2:**
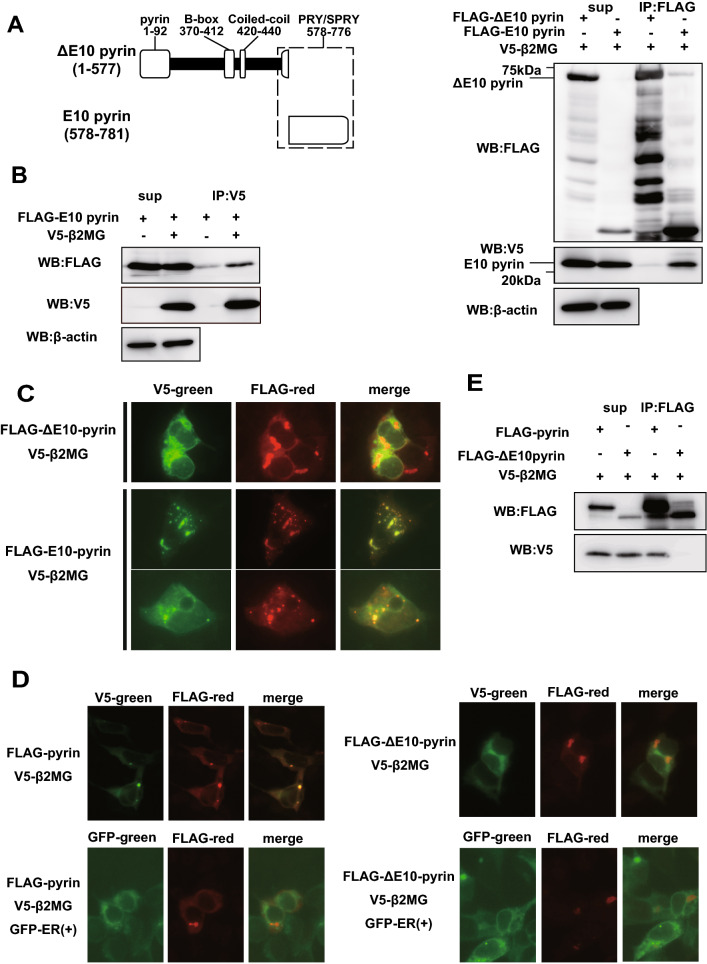
β2MG-binding site exists in the exon 10 of pyrin. (**A**) V5-β2MG and FLAG-tagged pyrin lacking exon10 (FLAG-Δ10 pyrin) or FLAG-tagged pyrin exon10 (FLAG-E10 pyrin) were expressed in Lenti-X 293T cells. The cells were lysed with lysis buffer and immunoprecipitated by FLAG Ab-conjugated agarose beads. Co-immunoprecipitation analysis reveals V5-β2MG binds to the pyrin exon10 region. (**B**) Lenti-X 293T cells expressing FLAG-E10 pyrin and V5-β2MG were subjected to immunoprecipitation analysis using V5 Ab-conjugated magnetic beads. The co-immunoprecipitation analysis confirms the interaction of the pyrin exon10 region and β2MG. (**C**) HEK293 cells expressing FLAG-ΔE10 or FLAG-E10 pyrin and V5-β2MG were subjected to immunofluorescence staining analysis. V5-β2MG (green) was colocalized with FLAG-E10 pyrin (red) in the speck-like structures in the perinuclear position in more than 90% of the overexpressed cells while no co-localization of V5-β2MG with FLAG-ΔE10 (red) was observed. (**D**) HEK293 cells expressing FLAG-pyrin or FLAG-ΔE10-pyrin with V5-β2MG were subjected to immunofluorescence analysis with or without GFP-tagged ER marker. FLAG-pyrin colocalizes with V5-β2MG forming speck-like structures. Neither FLAG-pyrin nor FLAG-ΔE10 is colocalized with the GFP-ER marker. (**E**) Lenti-X 293T cells expressing FLAG-pyrin or FLAG-ΔE10 with V5-β2MG were subjected to immunoprecipitation analysis using a hypotonic buffer to investigate protein–protein interactions in the cytoplasm. FLAG-pyrin, not FLAG-ΔE10, associates with V5-β2MG in the cytoplasm.

Because the *B2M* gene was identified not only by yeast two-hybrid screening using FL-pyrin bait but also using C-pyrin bait, we expected that β2MG can bind to the PRY/SPRY domain of pyrin. To further investigate the binding site for β2MG in pyrin molecule, we constructed the vectors which express FLAG-tagged pyrin lacking PRY/SPRY domain corresponding to exon 10 (ΔE10 pyrin) and pyrin PRY/SPRY domain (E10 pyrin) and transfected them with V5-β2MG expression vector into Lenti-X 293T cells. Although FLAG-E10 pyrin co-precipitated V5-β2MG, FLAG-ΔE10 pyrin did not (Fig. 2A). The reciprocal immunoprecipitation assay using anti-V5 Ab showed that FLAG-E10 pyrin was co-precipitated with V5-β2MG (Fig. 2B). Consistent with the data, immunofluorescence staining revealed that the overexpressed V5-β2MG was not colocalized with FLAG-ΔE10 pyrin but with FLAG-E10 in speck-like structures (Fig. 2C). These results indicate that pyrin interacts with β2MG via its PRY/SPRY domain.

To further examine the localization of pyrin–β2MG complex in HEK293T cells, we performed immunofluorescence staining using the endoplasmic reticulum (ER) marker. Pyrin and β2MG, which formed the speck-like structure in the perinuclear region, were not localized in the ER (Fig. 2D). We also performed immunoprecipitation analysis of Lenti-X 293T cells expressing FLAG-tagged full-length pyrin (FLAG-pyrin) or FLAG-ΔE10 pyrin with V5-β2MG using hypotonic cell lysis buffer, which extracts only cytoplasmic proteins. Like the results from the assays using the normotonic buffer, FLAG-pyrin co-precipitated V5-β2MG while FLAG-ΔE10 pyrin did not, indicating that pyrin and β2MG co-localize in the cytoplasm (Fig. 2E).

**Figure 3:**
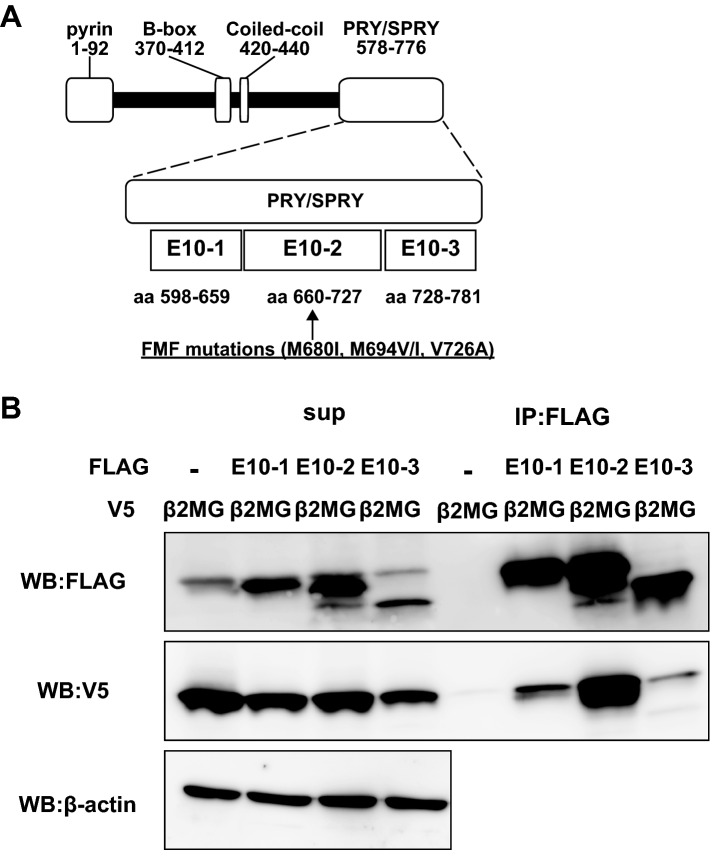
β2MG binds to the FMF mutation site. (**A**) Major *MEFV* mutations causing familial Mediterranean fever, M680I, M694I/V, and V726A, are concentrated in the middle portion of exon10 region (E10-2). (**B**) Lenti-X 293T cells expressing V5-β2MG and FLAG-tagged pyrin 10-1 (E10-1), 10-2 (E10-2), or 10-3 region (E10-3) were lysed with lysis buffer and subjected to co-immunoprecipitation analysis using FLAG Ab-conjugated agarose beads. Co-immunoprecipitation analysis reveals that β2MG binds to E10-2, which includes the hotspot of FMF-associated mutations.

FMF-associated mutations accumulate in the middle portion of the pyrin PRY/SPRY domain (Fig. 3A). Among them, M680I, M694V, M694I, and V726A are listed as the minimum set of clearly pathogenic sequence variants recommended to screen in the genetic diagnostic guidelines developed by Shinar et al.^23^. To elucidate the relationship between these mutations and β2MG binding, we constructed expression vectors for FLAG-tagged pyrin deletion mutants, pyrin E10-1 (aa 598–659), pyrin E10-2 (aa 660–727) and pyrin E10-3 (aa 728–781). These expression vectors were co-transfected with V5-tagged β2MG into Lenti-X 293T cells and subjected to co-immunoprecipitation analysis. As shown in Fig. 3B, all FLAG-tagged E10 pyrin mutants could co-precipitate V5-β2MG. However, FLAG-tagged pyrin E10-2, which includes the hotspot of FMF-associated mutations, co-precipitated V5-β2MG much more strongly as compared to other E10 mutants, suggesting the important role of mutations in the pyrin–β2MG interaction.

### β2MG–pyrin interaction recruits PSTPIP1 leading to the assembly of pyrin inflammasome

**Figure 4:**
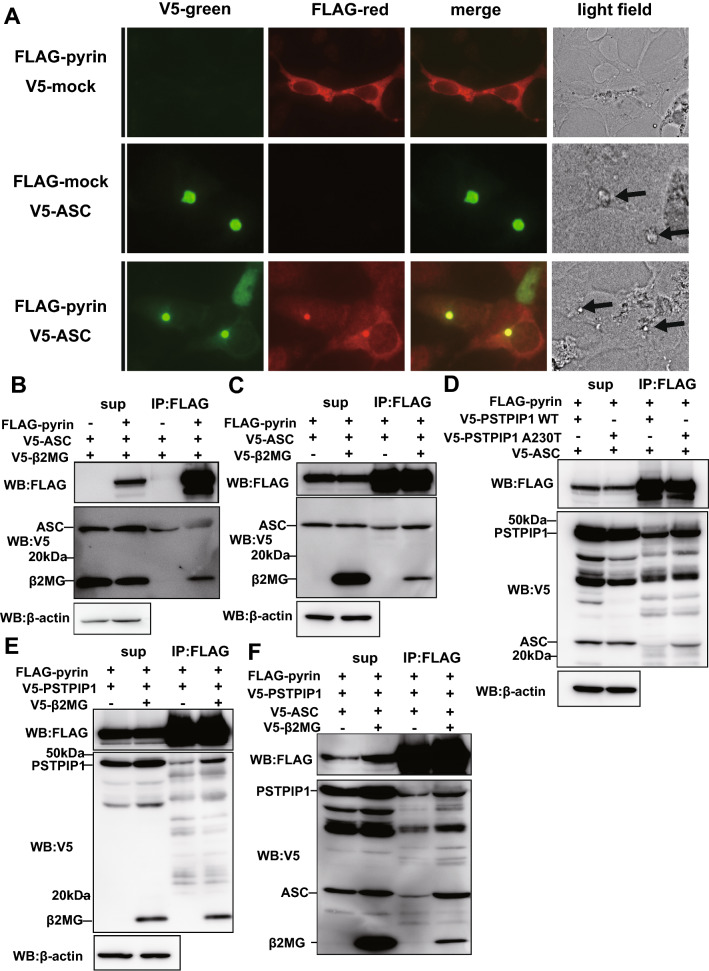
β2MG promotes PSTPIP1-mediated pyrin inflammasome formation. (**A**) HEK 293 cells expressing FLAG-pyrin and V5-tagged ASC were subjected to immunofluorescence staining analysis, which revealed co-localization of pyrin (red) and ASC (green) with the speck formation in more than 90% of the overexpressed cells. (**B**) Lenti-X 293T cells expressing V5-ASC and V5-β2MG with or without FLAG-pyrin were lysed with lysis buffer and subjected to co-immunoprecipitation analysis using FLAG Ab-conjugated agarose beads. Co-immunoprecipitation analysis reveals that pyrin does not associate with ASC. (**C**) Lenti-X 293T cells expressing FLAG-pyrin and V5-ASC with or without V5-β2MG were lysed with lysis buffer and subjected to co-immunoprecipitation analysis using FLAG Ab-conjugated agarose beads. Co-immunoprecipitation analysis reveals that pyrin–β2MG binding does not affect pyrin–ASC interaction. (**D**) Lenti-X 293T cells expressing FLAG-pyrin and V5-ASC with V5-tagged wild-type or A230T PSTPIP1 were lysed with lysis buffer and subjected to co-immunoprecipitation analysis by FLAG Ab-conjugated agarose beads. A230T PSIPTP1 binds to pyrin more strongly than the wild-type, leading to the enhanced recruitment of ASC. (**E**) Lenti-X 293T cells expressing FLAG-pyrin and V5-PSTPIP1 with or without V5-β2MG were lysed with lysis buffer and subjected to co-immunoprecipitation analysis using FLAG Ab-conjugated agarose beads. Co-immunoprecipitation analysis reveals that β2MG promotes pyrin–PSTPIP1 interaction (see also Supplementary Fig. S1A). (**F**) Lenti-X 293T cells expressing FLAG-pyrin, V5-PSTPIP1, and V5-ASC with or without V5-β2MG were subjected to co-immunoprecipitation analysis. β2MG promotes the pyrin–ASC interaction in the presence of PSTPIP1.

PSTPIP1, which was identified as a genetic cause of another autoinflammatory disorder, PAPA syndrome, has been shown to interact with the B-box/coiled-coil domain of pyrin^24^. Furthermore, PSTPIP binding has been shown to activate pyrin by unmasking the pyrin domain, promoting ASC-mediated oligomerization and inflammasome formation^14^. Thus, we next investigated whether pyrin–β2MG interaction affects pyrin–ASC and pyrin–PSTPIP1 interactions. As shown in Fig. 4A, when HEK293 cells overexpressing FLAG-tagged pyrin and V5-tagged ASC were subjected to immunofluorescence staining, FLAG-pyrin and V5-ASC were co-localized in the cytoplasm with the speck formation (*arrows in the light field images*), indicating pyrin–ASC interaction. To clarify the effect of pyrin–β2MG interaction on pyrin–ASC interaction, FLAG-tagged pyrin, and V5-tagged ASC expression vectors were transfected into Lenti-X 293T cells with or without V5-tagged β2MG expression vector. The co-immunoprecipitation analysis showed that pyrin did not interact with ASC (Fig. 4B) and β2MG overexpression did not induce pyrin–ASC interaction (Fig. 4C).

We next examined the effect of pyrin–β2MG interaction on pyrin–PSTPIP1 interaction. First, we investigated the interaction between pyrin, PSTPIP1, and ASC in the absence of β2MG overexpression by the co-immunoprecipitation analysis using FLAG-pyrin, V5-ASC expression vectors together with V5-tagged wild-type PSTPIP1 or one of the most common PAPA-associated PSTPIP1 mutation, A230T. FLAG-pyrin co-precipitated A230T PSTPIP1 drastically as compared to the wild-type PSTPIP1 (Fig. 4D), consistent with the previous study showing that A230T mutation induces its hyperphosphorylation and increases the affinity to pyrin^24^. Additionally, the pyrin–ASC interaction was also promoted by the mutation, indicating that the PSTPIP1 mutation increases the pyrin inflammasome formation (Fig. 4D). Then, FLAG-pyrin and V5-PSTPIP1 expression vectors were transfected into Lenti-X 293T cells with or without a V5-β2MG expression vector. Co-immunoprecipitation analysis revealed that β2MG overexpression drastically promoted the pyrin–PSTPIP1 interaction (Fig. 4E and Supplementary Fig. S1A).

To assess whether β2MG promotes ASC recruitment, we have overexpressed V5-β2MG together with pyrin, PSTPIP1, and ASC in the cells, and investigated the change of ASC recruitment by the β2MG-mediated enhancement of pyrin–PSTPIP1 interaction. As a result, β2MG overexpression promoted ASC recruitment (Fig. 4F). The ability is PSTPIP1-dependent because the induction of ASC recruitment was not observed without PSTPIP1 overexpression (Fig. 4C).

### β2MG and pyrin interaction leads to caspase-1 activation and IL-1β secretion

**Figure 5:**
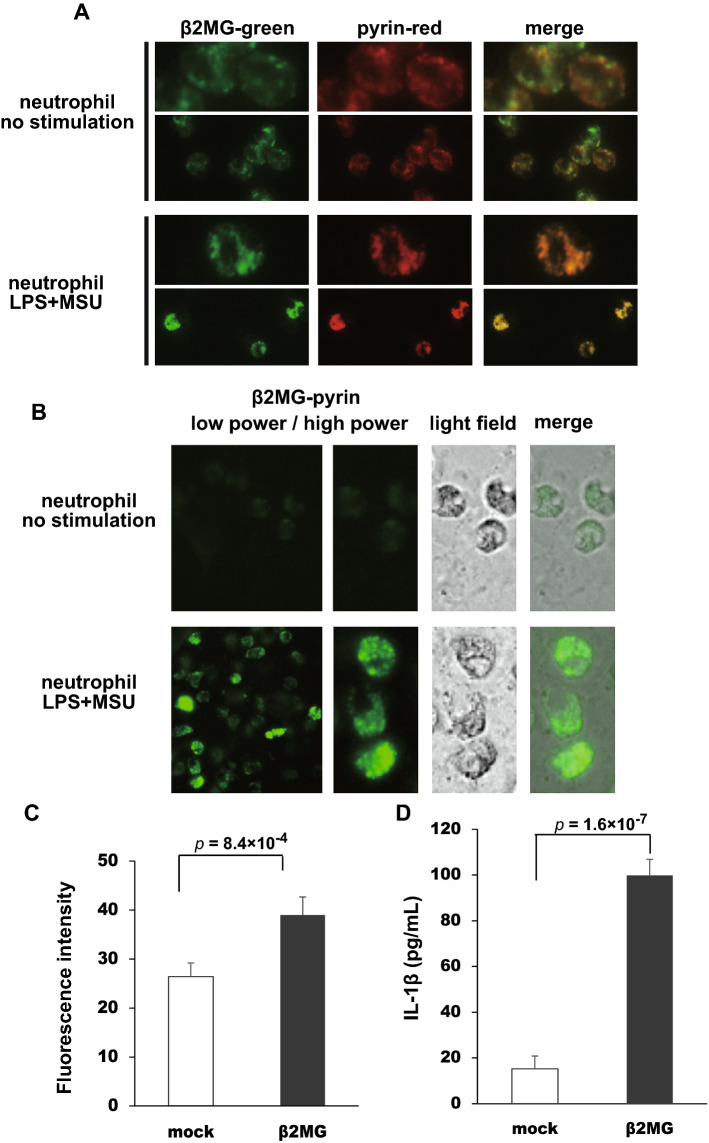
The assembly of pyrin inflammasome leads to caspase-1 activation and IL-1β secretion. (**A**) Human neutrophils stimulated with LPS and MSU crystals were subjected to immunofluorescence staining analyses. Pyrin (red) and β2MG (green) are co-localized in cytoplasm, with forming speck, in the stimulated neutrophils while they are separately localized in cytoplasm and perinuclear granules, respectively, in the unstimulated neutrophils. (**B**) The interaction between pyrin and β2MG was investigated using proximity ligation assay. The microscopic images showed fluorescence signals indicating the pyrin–β2MG interaction in the cytosol of human neutrophils stimulated with LPS and MSU crystals. (**C**) THP-1 cells transfected with mock or V5-β2MG expression vector were subjected to FLICA assay. The overexpression of β2MG in THP-1 cells leads to the stronger capsase-1 activation. Values are the means of nine experiments ± standard error of mean (SEM). (**D**) THP-1 cells transfected with mock or V5-β2MG expression vector were cultured for 24 h, and cell supernatants were subjected to IL-1β ELISA. The overexpression of β2MG in THP-1 cells increases the secretion of IL-1β. Values are the means of six experiments ± SEM.

To confirm the localization of endogenous pyrin and β2MG, we examined human neutrophils isolated from the peripheral blood of healthy donors and stained with anti-pyrin and anti-β2MG Abs. β2MG staining was not co-localized with pyrin staining in neutrophils without any stimulation (Fig. 5A, upper panels). Because interaction between neutrophils and monosodium urate (MSU) crystals has been shown to elicit various responses, including IL-1β secretion^25^, we next investigated their localization in the stimulated neutrophils. Interestingly, β2MG was co-localized with pyrin in neutrophils stimulated with LPS and MSU in the speck-like structures (Fig. 5A, lower panels), suggesting that the pyrin–β2MG interaction is induced by neutrophil activation. To further confirm the interaction of β2MG and pyrin in neutrophils, we performed a proximity ligation assay of these proteins in unstimulated or stimulated neutrophils. As shown in Fig. 5B, β2MG and pyrin were co-localized in the speck-like structure.

We also performed a fluorescent-labeled inhibitor of caspases (FLICA) assay to investigate whether pyrin–β2MG interaction leads to caspase-1 activation. We overexpressed V5-β2MG in a human monocyte-like cell line, THP-1 cells, and evaluated the caspase-1 activity by FLICA and IL-1β secretion by enzyme-linked immuno-sorbent assay (ELISA). As shown in Fig. 5C, β2MG overexpression strengthened the luminescence intensity, indicating increased caspase-1 activation. Moreover, β2MG overexpression also increased the IL-1β secretion (Fig. 5D). These results suggest that β2MG functions as a pyrin-binding protein inducing PSTPIP1-mediated pyrin inflammasome formation.

### Caspase-1 p20 subunit inhibits the association of β2MG with pyrin

**Figure 6:**
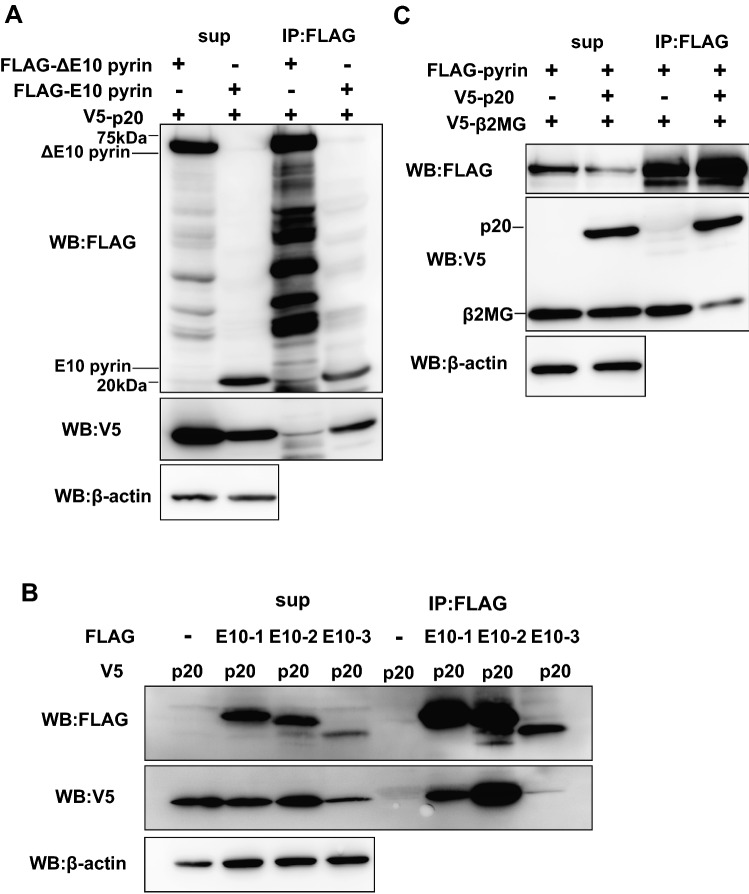
Caspase-1 p20 subunit interacts with pyrin in a manner that competes with β2MG. (**A**) FLAG-Δ10 pyrin and FLAG-E10 pyrin were expressed with V5-tagged caspase-1 p20 subunit (V5-p20) respectively in Lenti-X 293T cells. They were lysed with lysis buffer and subjected to co-immunoprecipitation analysis using FLAG Ab conjugated agarose. V5-p20 binds to pyrin exon10 region. (**B**) Lenti-X 293T cells expressing each pyrin E10-1, E10-2 and E10-3 region with V5-p20 were lysed with lysis buffer. Co-immunoprecipitation analysis using FLAG Ab conjugated agarose reveals caspase-1 p20 binds to the pyrin E10-2 region, the same as β2MG. (**C**) Lenti-X 293T cells expressing FLAG-pyrin and V5-β2MG with or without V5-p20 were lysed with lysis buffer and subjected to co-immunoprecipitation analysis using FLAG Ab conjugated agarose. Co-immunoprecipitation analysis reveals caspase-1 p20 subunit binds to pyrin competitively with β2MG and inhibits pyrin–β2MG binding.

The activation of caspase-1 is caused by the autocleavage of pro-caspase-1, which leads to the formation of active caspase-1 p10/p20 tetramer^26^. It is a pivotal process for IL-1β and IL-18 production after the assembly of the pyrin inflammasome. On the other hand, p20 and p10 have been shown to interact with the PRY/SPRY domain of pyrin directly, resulting in the inhibitory effect on IL-1β production^18^. Thus, we hypothesized that the binding of β2MG and caspase-1 to pyrin may compete on the pyrin PRY/SPRY domain. We constructed the expression vectors for V5-tagged caspase-1 p20 and transfected them into the Lenti-X 293T cells with the expression vectors for FLAG-tagged ΔE10 or E10 pyrin. Co-immunoprecipitation analysis revealed that caspase-1 p20 interacts with the PRY/SPRY domain of pyrin as shown in the previous report (Fig. 6A)^18^. To investigate the relationship between the FMF-associated pyrin mutations and caspase-1 binding, we co-transfected the vectors for FLAG-tagged pyrin deletion mutants, pyrin E10-1, E10-2 or E10-3 with V5-tagged p20 into Lenti-X 293T cells and subjected them to co-immunoprecipitation analysis. As is the case with β2MG, FLAG-tagged E10-2, which includes the hotspot of FMF-associated mutations, co-precipitated V5-p20 best among the pyrin deletion mutants (Fig. 6B). To elucidate whether caspase-1 has an inhibitory effect on pyrin–β2MG interaction, FLAG-tagged pyrin and V5-tagged β2MG expression vectors were co-transfected into Lenti-X 293T cells with or without V5-tagged caspase-1 p20 expression vector. Co-immunoprecipitation analysis revealed that overexpression of caspase-1 p20 inhibits the interaction between pyrin and β2MG (Fig. 6C). These results suggest that p20 acts as an inhibitor for β2MG-mediated promotion of pyrin inflammasome formation and suppresses the excess inflammation via a negative feedback mechanism.

### Pyrin M694V mutation changes the balance of affinities to β2MG and caspase-1 p20

**Figure 7:**
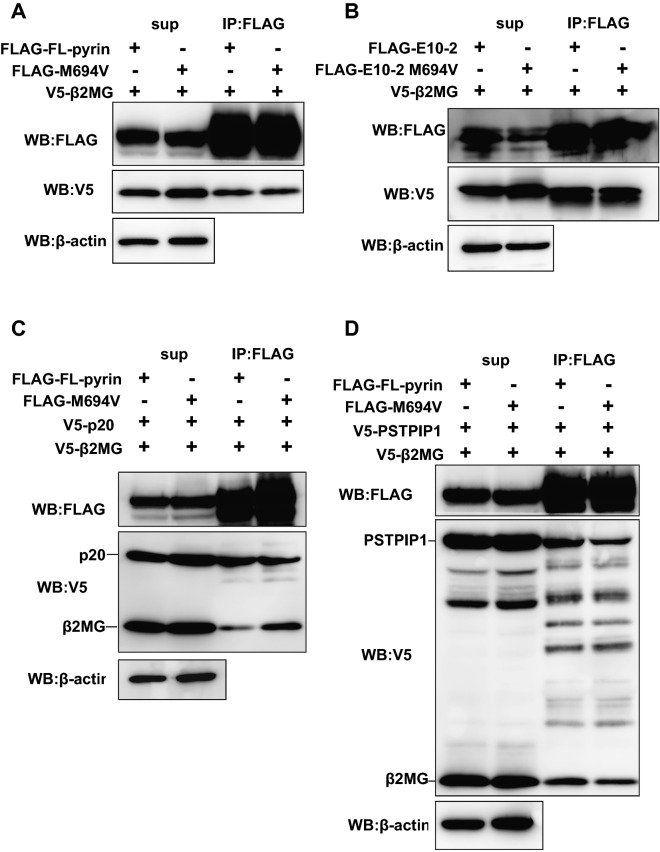
M694V mutation of pyrin weakens the competitive binding of p20 and promotes the recruitment of PSTPIP1 in the presence of β2MG. (**A**) Lenti-X 293T cells expressing FLAG-tagged full-length (FL) pyrin or M694V pyrin with V5-β2MG were lysed with lysis buffer and subjected to co-immunoprecipitation analysis using FLAG Ab-conjugated agarose. M694V mutation does not affect pyrin–β2MG affinity. (**B**) Lenti-X 293T cells expressing FLAG-tagged pyrin deletion mutant E10-2 or M694V-mutated E10-2 with V5-β2MG were lysed with lysis buffer and subjected to co-immunoprecipitation analysis by FLAG Ab-conjugated agarose beads. M694V mutation does not affect the affinity of E10-2 for β2MG. (**C**) Lenti-X 293T cells expressing FLAG-tagged FL- or M694V pyrin and V5-β2MG with or without V5-p20 were lysed with lysis buffer and subjected to co-immunoprecipitation analysis using FLAG Ab-conjugated agarose. Co-immunoprecipitation analysis reveals that M694V pyrin is prone to bind β2MG in spite of the presence of p20 (see also Supplementary Fig. S1B). (**D**) Lenti-X 293T cells expressing FLAG-tagged FL- or M694V pyrin with V5-PSTPIP1 and V5-β2MG were lysed with lysis buffer and subjected to co-immunoprecipitation analysis using FLAG Ab-conjugated agarose beads. Co-immunoprecipitation analysis shows no difference in the recruitment of PSTPIP1 between the wild type and M694V pyrin (see also Supplementary Fig. S1C).

Homozygous M694V mutation can cause severer forms of FMF and AA amyloidosis in higher prevalence^27^. To elucidate whether M694V mutation affects β2MG affinity for pyrin, we constructed expression vectors for FLAG-tagged full-length pyrin and E10-2 fragment with M694V mutation and compared the β2MG affinity between for M694V-mutated pyrin or E10-2 fragment and for the wild type by co-immunoprecipitation assay. As shown in Fig. 7A,B the M694V mutation did not make any impact on the β2MG affinity in the absence of caspase-1 p20 overexpression. Because our data showed that caspase-1 p20 inhibits the association of β2MG with pyrin, we next investigated the effect of M694V mutation on inhibition of pyrin–β2MG interaction by p20. Unlike wild-type pyrin, M694V-mutated pyrin co-precipitated β2MG even in the presence of p20, suggesting that pyrin M694V mutation weakened negative feedback on PSTPIP1-mediated pyrin inflammasome formation by caspase-1 p20 (Fig. 7C and Supplementary Fig. S1B).

Finally, we examined whether pyrin M694V mutation affects PSTPIP1 recruitment by co-immunoprecipitation analysis. As a result, M694V-mutation itself did not affect the pyrin–PSTPIP1 interaction in the presence of β2MG (Fig. 7D and Supplementary Fig. S1C). These findings suggest that pyrin M694V mutation leads to the overproduction of pyrin inflammasome not by promoting β2MG-induced PSTPIP1 recruitment but by reduction of the inhibitory effect of caspase-1 p20 on pyrin–β2MG interaction.

## Discussion

Although the function of pyrin protein, encoded by the *MEFV* gene, has been investigated by many researchers since *MEFV* was identified as the responsible gene for FMF, it has not been completely understood how *MEFV* mutations affect the function of pyrin. In this study, we identified β2MG as a protein that binds to the pyrin PRY/SPRY domain, which was competitively blocked by caspase-1 p20 subunit. β2MG–pyrin interaction leads to the recruitment of PSTPIP1, a crucial component of pyrin inflammasome. Pyrin M694V mutation strengthened the β2MG–pyrin interaction.

Recently, several papers have shown that RhoA GTPase indirectly regulates pyrin inflammasome activation. RhoA-dependent serine/threonine protein kinases PKN1 and PKN2 phosphorylate pyrin at S208 and S242 residues, which are located between pyrin and B-box domains, and 14-3-3 protein traps the phosphorylated pyrin, preventing pyrin from inflammasome formation^28^. They also elucidated that some bacterial toxins, such as TcdB from *Clostridium difficile*, inhibit the activity of RhoA, leading to a decrease of PKN1/2 activities and pyrin phosphorylation, which in turn make the pyrin dissociate from 14-3-3 and facilitate active pyrin inflammasome formation. These findings are supported by the reports on the dominantly inherited disorder called pyrin-associated autoinflammation with neutrophilic dermatosis (PAAND)^29,30^. In patients with PAAND, pyrin S242A or G244L mutation cause a decrease of PKN1/2-dependent pyrin phosphorylation and binding to 14-3-3, triggering the spontaneous pyrin activation. Although this RhoA-dependent pyrin activation model well describes the physiological function of pyrin as a surveillance protein against bacterial toxins without directly recognizing the pathogen-associated molecular patterns, it is difficult to explain the pathological significance of this model since FMF-associated mutations are clustered in PRY/SPRY domain of pyrin. Considering that RhoA-dependent pyrin activation is observed in murine macrophage whose pyrin lacks PRY/SPRY domain^31^ and that there are differences in clinical symptoms between FMF and PAAND, it is plausible that PRY/SPRY domain of human pyrin has a function for inflammasome formation in a certain manner other than the RhoA-dependent pathway. As expected, we have discovered the new PRY/SPRY domain-binding regulator, β2MG, for pyrin inflammasome formation in this study.

β2MG is ubiquitously expressed mainly as a component of MHC class I or CD1 molecules, contributing to antigen presentation. On the other hand, it has been reported that in neutrophil two-thirds of β2MG molecules are localized in granules, including gelatinase granules and specific granules^32^. The function and binding proteins of β2MG contained in granules of neutrophil had not been known before we identified β2MG as a pyrin-binding protein in this study. Under normal circumstances, intragranular β2MG and cytoplasmic pyrin never interact because of their different localizations. This raises the question of where and how pyrin interacts with β2MG in the cell. When neutrophils phagocytose foreign bodies resistant to phagosomal digestion such as MSU crystals, phagosomes containing them fuse with granules, eventually rupture, and are unable to digest its contents^33,34^. This event is called “phagosomal destabilization” and results in the cytosolic distribution of intragranular proteins. Although the phagosome destabilization has been argued mainly for the activation of NLRP3 inflammasome so far, it can also cause the pyrin inflammasome activation as these two inflammasomes can be activated by the same stimulation^21,35,36^. Thus, we suspect that the pyrin–β2MG interaction can be occurred by this phagosomal destabilization before pyrin inflammasome formation. Consistent with this idea, β2MG was not co-localized with pyrin in human neutrophils without stimulation while their co-localization was observed in neutrophils stimulated with LPS and MSU in the speck-like structures. Pyrin can be considered to function as a cytosolic sensor for phagosomal destabilization by detecting β2MG.

FMF is, as the name suggests, prevalent in Mediterranean descendants and the selective advantage for the *MEFV* mutation carrier is speculated^37^. A recent study showed that FMF mutant pyrin interacts less firmly with a virulence factor YopM of *Yersinia pestis*, which is an intracellular parasite, than the wild-type pyrin, thereby attenuating YopM-induced interleukin-1β suppression^38^. Thus, FMF mutations positively selected in Mediterranean populations increase resistance to *Y. pestis*. Considering other intracellular parasites such as *Mycobacterium tuberculosis* and *Listeria monocytogenes* are known to tolerate or escape from the phagolysosomal digestion of macrophages^39^, not only crystal structures like MSU but also such pathogenic microorganisms can cause the phagosomal destabilization in neutrophils. Based on our hypothesis, the selective advantage for the *MEFV* mutation carrier can partly rely on the expected mechanism that β2MG can be released into the cytoplasm by the trigger due to infection and that following pyrin inflammasome activation may have a pivotal role in the control of infection.

PSTPIP1 harboring PAPA syndrome-associated mutations (A230T and E250Q) presents a reduced affinity for PEST (rich in proline [P], glutamic acid [E], serine [S], and threonine [T])-type protein tyrosine phosphatase (PTP-PEST), resulting in increased phosphorylation of PSTPIP1. Subsequently, the hyperphosphorylated PSTPIP1 strongly binds to pyrin, leading to excessive formation of pyrin inflammasome^24^. Although the importance of PSTPIP1 in pyrin inflammasome formation had been elucidated, it was unknown what causes pyrin–PSTPIP1 interaction other than the PAPA syndrome-associated PSTPIP1 mutations. Our findings suggest that the β2MG binding to pyrin promotes pyrin–PSTPIP1 interaction, which leads to pyrin inflammasome formation. It indicates the possibility that there exists a RhoA-independent pyrin activation pathway.

The present study also suggests the important role of interaction between pyrin and caspase-1 p20 subunit for the control of pyrin inflammasome activation. There are conflicting reports on pyrin–caspase-1 interaction. One study showed all full-length and p20, p10 subunits of caspase-1 interacted with the pyrin PRY/SPRY domain and that the FMF-associated mutation of pyrin decreased its affinity for caspase-1^18^. On the contrary, another paper reported that the interaction of caspase-1 with the pyrin PRY/SPRY domain with the M694V mutation is equally strong as the one observed with the wild-type, suggesting that the mutation does not affect the binding of pyrin to caspase-1^20^. In our study, the caspase-1 p20 subunit strongly bound to the pyrin PRY/SPRY domain, inhibiting pyrin–β2MG interaction, and M694V mutation modified this antagonism toward the interaction with β2MG. Thus, we consider that the FMF-associated pyrin mutation can cause stronger inflammasome activation via its decreased affinity for caspase-1 and increased interaction with β2MG.

To further strengthen the validity of our findings on the role of M694V mutation in developing FMF, it could be helpful to compare the protein–protein interactions and pyrin inflammasome activation described above between FMF patients with M694V mutation and healthy controls. Unfortunately, as a limitation of this study, it is difficult for us to obtain samples from such patients because FMF patients harboring homozygous M694V mutations are extremely rare in Japan. Although the most frequent FMF mutations seen on the Mediterranean coast are M694V, heterozygotes of E148Q and M694I are the most common in Japan, and M694V has not been found so far. This issue will be an agenda for future studies and could be solved by international joint research.

Another limitation of this study is that we do not confirm the protein–protein interaction network shown by the overexpression experiments in primary neutrophils. Although it is technically difficult to show the protein–protein interaction using primary neutrophils, we should overcome this in future studies.

Based on our findings, here we propose a novel hypothesis for FMF pathogenesis. β2MG functions as a pyrin ligand inducing pyrin inflammasome formation and subsequent production of active caspase-1 p20 and p10 subunits, followed by blocking of the β2MG–pyrin interaction by p20 in a negative feedback manner. Pyrin M694V mutation disrupts the negative feedback by caspase-1 p20 subunit, resulting in the promotion of pyrin inflammasome formation and the vicious cycle of inflammation (Supplementary Fig. S2).

## Methods

### Yeast two-hybrid library screening

Yeast two-hybrid screening was performed using the Matchmaker Gold Two-hybrid System (Clontech Laboratories, Palo Alto, CA). To construct bait plasmids, full-length human pyrin (FL-pyrin) and the C-terminal region of human pyrin (C-pyrin; aa 598–781) was cloned in frame with the GAL4 DNA-binding domain of pGBKT7 vector. The yeast strain AH109 was transformed with pGBKT7-FL-pyrin and pGBKT7-C-pyrin, respectively. As prey plasmids, human leukocyte cDNA library was inserted downstream of the GAL4 activation domain of pACT2 vector (Clontech Laboratories), and then the yeast strain Y187 was transformed with the plasmids. After mating the AH109 and Y187 transformants, they were spread on plates with high selectivity medium (synthetic drop-out medium without adenine, histidine, tryptophan, and leucine). After identifying the positive colonies by β-galactosidase activity, cDNAs inserted in the prey vector were amplified by colony-direct PCR. The positive inserts were sequenced and analyzed using the BLAST program (National Center for Biotechnology Information, Bethesda, MD).

### Abs

Anti-FLAG monoclonal and polyclonal Abs were obtained from Sigma-Aldrich. Anti-V5 monoclonal Ab was obtained from Invitrogen. Anti-pyrin rabbit polyclonal Ab, anti-pyrin mouse monoclonal Ab (4E6), and anti-β2MG mouse monoclonal Ab were from Sigma-Aldrich.

### MSU crystal preparation

MSU crystals were prepared by the method described by Seegmiller et al.^40^. Briefly, uric acid (Sigma-Aldrich) was dissolved in boiling water, and the pH of the solution was adjusted to 7.2 by adding NaOH. Then the solution was cooled slowly while stirring at room temperature until MSU crystals precipitated. The crystals were washed with ethanol, autoclaved, suspended in PBS at a concentration of 10 mg/ml, and then sonicated to obtain rod-shaped crystals.

### Cell culture

HEK293 cells and Lenti-X 293T (Clontech Laboratories) were cultured at 37 °C in a humidified atmosphere of 5% CO_2_ in DMEM medium containing 10% FBS, 100 U/ml penicillin, and 100 μg/ml streptomycin. THP-1 cells were cultured at 37 °C in a humidified atmosphere of 5% CO_2_ in RPMI1640 medium containing 10% FBS, 100 U/ml penicillin, and 100 μg/ml streptomycin.

Human neutrophils were isolated from the peripheral blood of healthy donors by centrifugation using Polymorphprep (Abbot diagnostic technologies AS) as a manufacturer’s instruction. Purified neutrophils were resuspended in RPMI medium with 10% FBS and cultured in 35-mm dishes at the concentration of 2 × 10^5^ cells per dish. Neutrophils were primed with 500 ng/ml *Salmonella enterica* lipopolysaccharide (LPS; Sigma-Aldrich) for 3 h and then stimulated with 200 μg/ml MSU crystals for 2 h, which were subjected to immunofluorescence analyses. The study protocol is approved by the ethics committee of Yokohama City University Hospital (B100701027, B191000004), and informed consent was obtained from the healthy controls. The study was conducted based on the Declaration of Helsinki.

### Transfection

For HEK293 cells, plasmid transfection was performed by lipofection using lipofectamine LTX (Invitrogen) for immunofluorescence analyses. For Lenti-X 293T cells, plasmid transfection was performed using calcium phosphate method and for immunoprecipitation assays and Western blotting. For THP-1 cells, plasmid transfection was performed by lipofection using lipofectamine LTX and by electroporation using Nucleofector 2 (Lonza) according to manufacturer’s instructions.

### Immunoprecipitation assay

Lenti-X 293T cells (2.0–4.0 × 10^6^ cells) transfected with the expression plasmids were lysed with the 500 μl of lysis buffer (20 mM Tris–HCl (pH 6.0), 120 mM NaCl, 5% glycerol, and 0.5% Triton X-100). After clarification by centrifugation at 20,000×*g* for 30 min, each 20 μl of these lysates was used as supernatant, and the remaining were incubated with 5 μl of agarose beads conjugated with anti-FLAG Ab (Sigma-Aldrich) or 10 μl of magnet beads conjugated with anti-V5 Ab (Medical and Biological Laboratories) for 1 h at 4 °C. After extensive washing with the lysis buffer, the immunocomplexes were solubilized by adding SDS sample buffer to the beads and subjected to Western blot analysis using a chemiluminescence ECL system (Amersham Biosciences). Anti-FLAG and anti-V5 Abs were diluted to 1:5000 for Western blot analyses. β-Actin was detected as a loading control using 1:10,000 dilution of Direct-Blot HRP anti-β-actin Antibody (BioLegend).

To examine cytoplasmic protein–protein interactions, we lysed cells with 500 μl of the hypotonic lysis buffer (20 mM Tris–HCl (pH 6.0), 10 mM KCl, 1.5 mM MgCl_2_, 5% glycerol) and 20 times of syringing with 27G needle. After centrifugation at 1000×*g* for 10 min, NaCl was added to reach 120 mM and re-clarified by centrifugation at 20,000×*g* for 30 min. The supernatant was subjected to immunoprecipitation analysis.

We performed each experiment at least three times to confirm reproducibility. Each data in the figures show a representative of them.

### Immunofluorescence staining

HEK293 cells transfected with expression plasmids were cultured on human type I collagen-coated glass chamber slides (BD biosciences). After washed with PBS, they were fixed with 2% paraformaldehyde in PBS for 15 min at room temperature and then permeabilized with PBS containing 0.5% Triton X-100 for 10 min. After blocking with PBS containing 10% BSA, the cells were treated with appropriate primary Abs (rabbit anti-FLAG polyclonal Ab and mouse anti-V5 monoclonal Ab) for 1 h at 37 °C in a moist chamber, washed with PBS containing 0.05% Tween 20, and incubated with secondary Abs [Alexa Flour 488 donkey anti-mouse IgG and Alexa Fluor 555 goat anti-rabbit IgG Abs (Amersham Biosciences)]. After washing, the samples were observed under a fluorescence microscope, BZ9000 (Keyence). For labeling ER with GFP, we used CellLight™ ER-GFP, BecMam 2.0 (Thermo Fisher Scientific) according to the manufacturer’s protocols.

Neutrophils with or without stimulation were collected by centrifugation at 500×*g* for 10 min. After washed with PBS, fixed, permeabilized, and blocked as described above, the cells were treated with primary Abs (rabbit anti-pyrin polyclonal Ab and mouse anti-β2MG Ab) diluted to 1:1000. After washing, the cells were incubated with the secondary Abs. We performed each experiment at least three times to confirm reproducibility. Each data in the figures show a representative of them.

### FLICA assay

FLICA assay was performed using Pyroptosis/Caspase-1 Assay Kit (ImmunoChemistry Technologies) according to the manufacturer’s protocols. Briefly, THP-1 cells transfected with mock or β2MG expression vector were incubated with a green fluorescent carboxyfluorescein dye-labeled caspase-1 inhibitor (FAM-YVAD-FMK) for 1 h at 37 °C and washed with cellular wash buffer for three times. Then samples were suspended in cellular wash buffer, and fluorescence intensity was measured on a microplate reader (Powerscan^®^HT; DS Pharma Biomedical) set at 485 nm excitation and 528 nm emission.

### ELISA

The IL-1β concentration of the culture supernatant was measured using an ELISA kit (Sigma-Aldrich) according to the manufacturer’s protocols. The absorbance at 450 nm was read with the microplate reader (Powerscan^®^HT; DS Pharma Biomedical).

### Proximity ligation assay

Proximity ligation assay was performed using Duolink^®^ proximity ligation assay kit (Merck). Anti-pyrin mouse monoclonal Ab was conjugated with PLUS nucleotide probe, and anti-β2MG mouse monoclonal Ab was conjugated with MINUS nucleotide probe, respectively. Unstimulated and stimulated neutrophils were subjected to the proximity ligation assay according to the manufacturer’s protocols.

### Statistical analysis

Pared samples data were analyzed using pared *t* test. *P* values less than 0.05 were considered to be statistically significant.

## Supplementary Information

Supplementary Figures.

## Data Availability

The datasets analyzed during the current study are available from the corresponding author on reasonable request.
