# Supplementary material for: The PRY/SPRY domain of pyrin/TRIM20 interacts with β2-microglobulin to promote inflammasome formation
Source: Sci Rep. 2021 Dec 8;11:23613. doi: 10.1038/s41598-021-03073-6 (PMC8654936; doi:10.1038/s41598-021-03073-6)

The PRY/SPRY Domain of Pyrin/TRIM20 Interacts with β_2-Microglobulin to Promote Inflammasome Formation

Sei Samukawa, Ryusuke Yoshimi*, Yohei Kirino & Hideaki Nakajima

Department of Stem Cell and Immune Regulation, Yokohama City University Graduate School of Medicine, Yokohama 236-0004, Japan.

*Corresponding author: Ryusuke Yoshimi, MD, PhD
E-mail: yoshiryu@med.yokohama-cu.ac.jp

Supplementary Information

Supplementary Figure S1

Densitometric analysis of immunoprecipitations.

Supplementary Figure S2

Schematic view of possible mechanisms of pyrin inflammasome regulation and role of *MEFV* mutation in FMF.

Appendix

The full images of Western blot analyses.

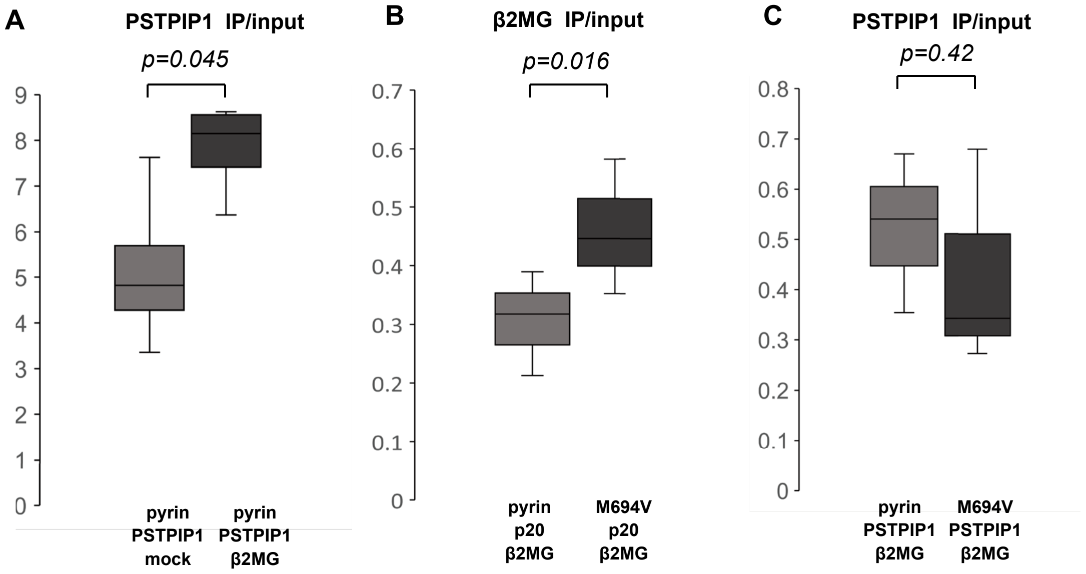

Supplementary Figure S1. Densitometric analysis of immunoprecipitations. Immunoprecipitate levels of PSTPIP1 by pyrin with or without β2MG (A), β2MG by pyrin or M694V mutant with p20 (B), and PSTPIP1 by pyrin or M694V with β2MG (C) were analyzed by densitometry. (A) β2MG significantly promotes pyrin and PSTPIP1 interaction (n = 4; see also Figure 5E). (B) M694V mutation of pyrin significantly weakens competitive inhibition of the pyrin-β2MG interaction by caspase-1 p20 (n = 3; see also Figure 7C). (C) M694V mutation of pyrin does not affect PSTPIP1 recruitment by β2MG (n = 3; see also Figure 7D).

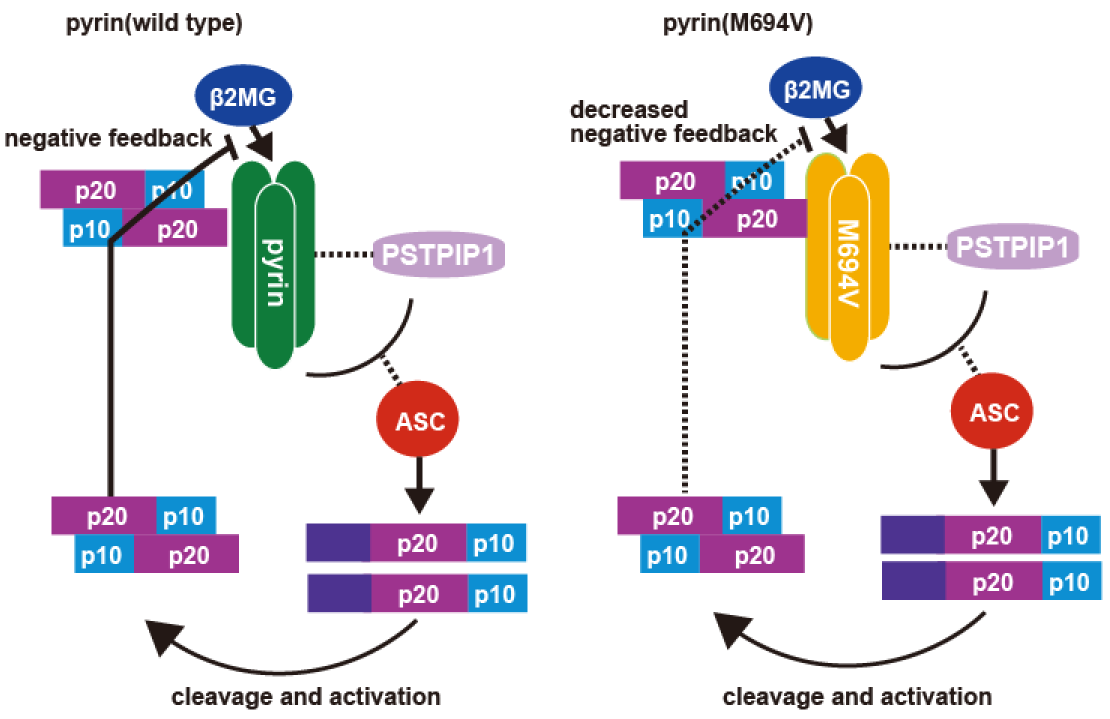

Supplementary Figure S2. Schematic view of possible mechanisms of pyrin inflammasome regulation and role of *MEFV* mutation in FMF. The pyrin-β2MG interaction recruits PSTPIP1 and subsequently ASC, leading to pyrin inflammasome formation. The pyrin inflammasome activates caspase-1, and then the active caspase-1 subunit p20 suppresses the pyrin-β2MG interaction in a negative feedback manner. M694V mutation of pyrin weakens the negative feedback by p20, resulting in the excessive formation of pyrin inflammasome.

Appendix

Figure 1B

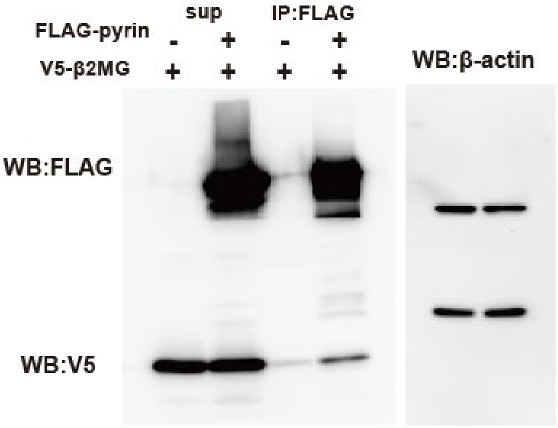

Figure 1C

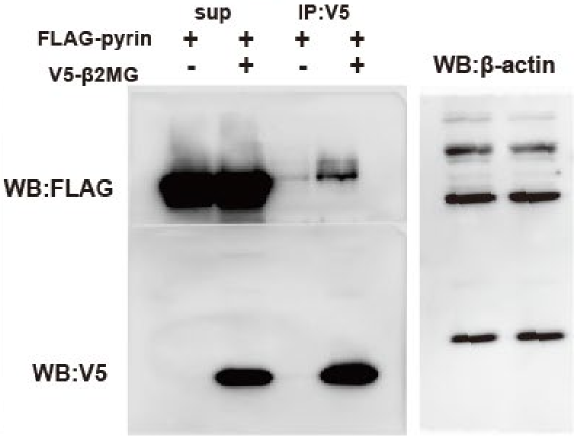

Figure 2A

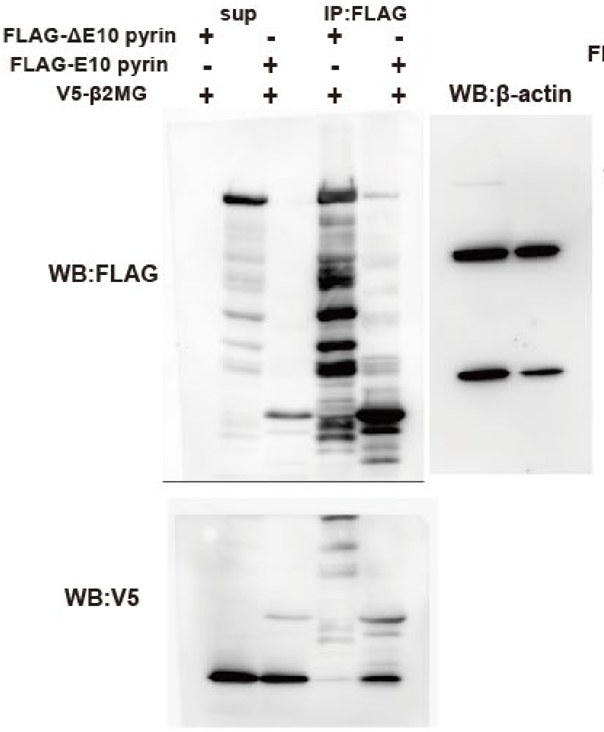

Figure 2B

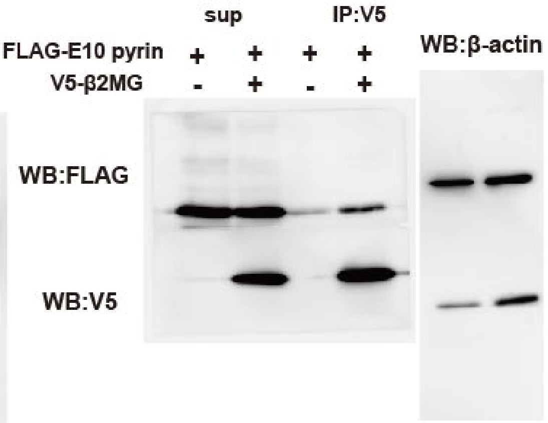

Figure 2E

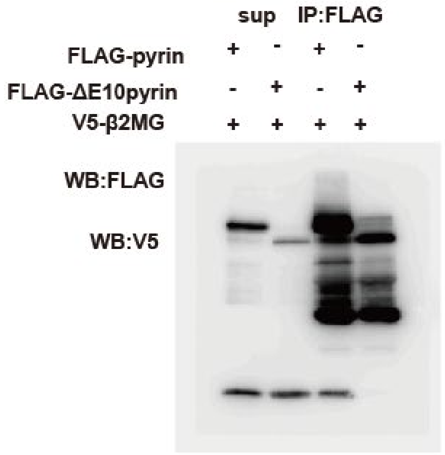

Figure 3B

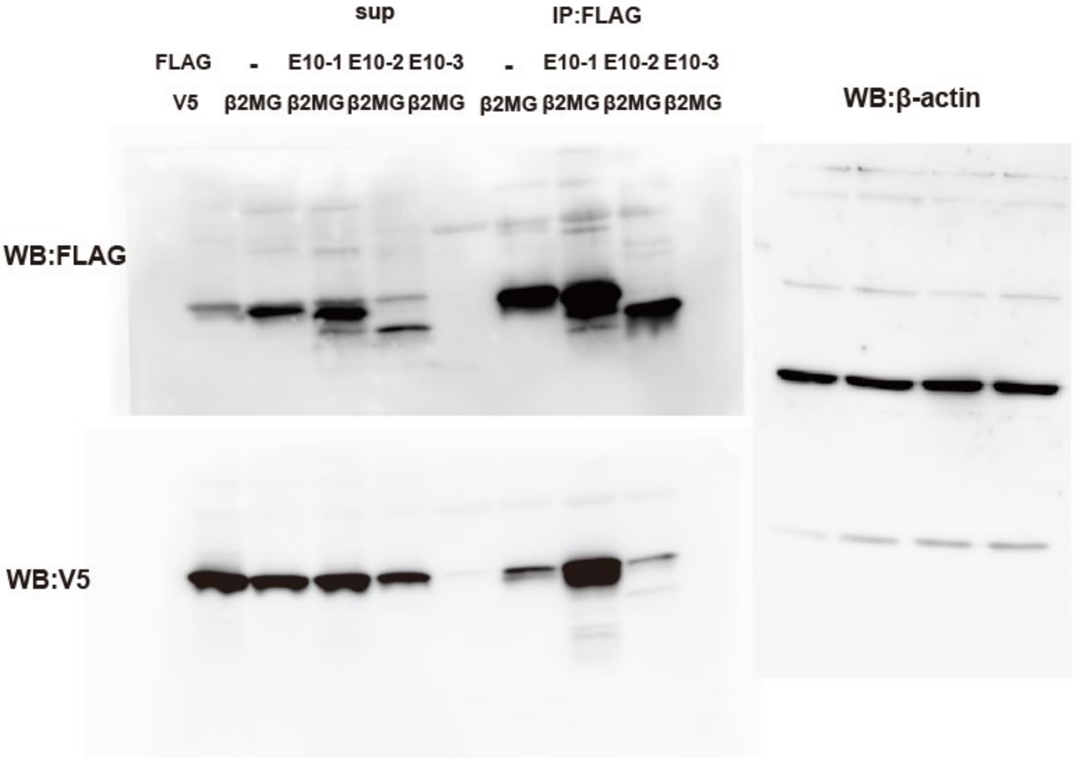

Figure 4B

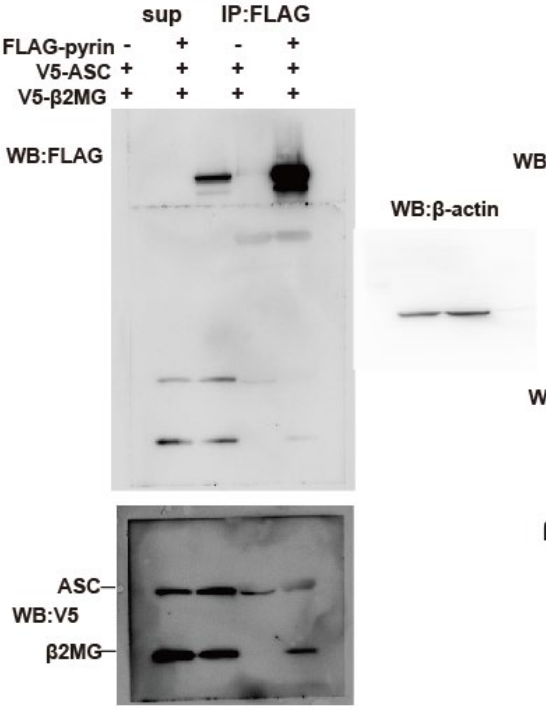

Figure 4C

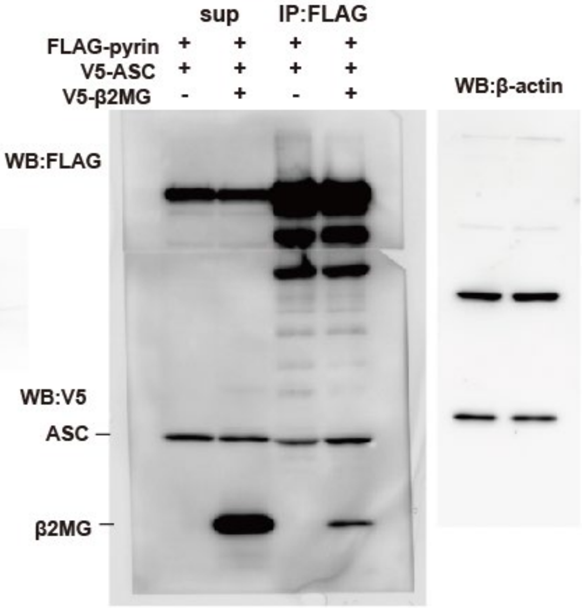

Figure 4D

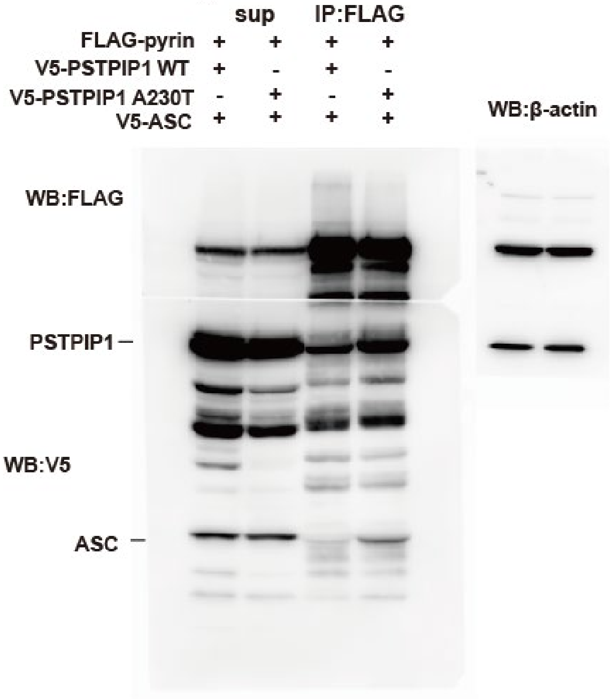

Figure 4E

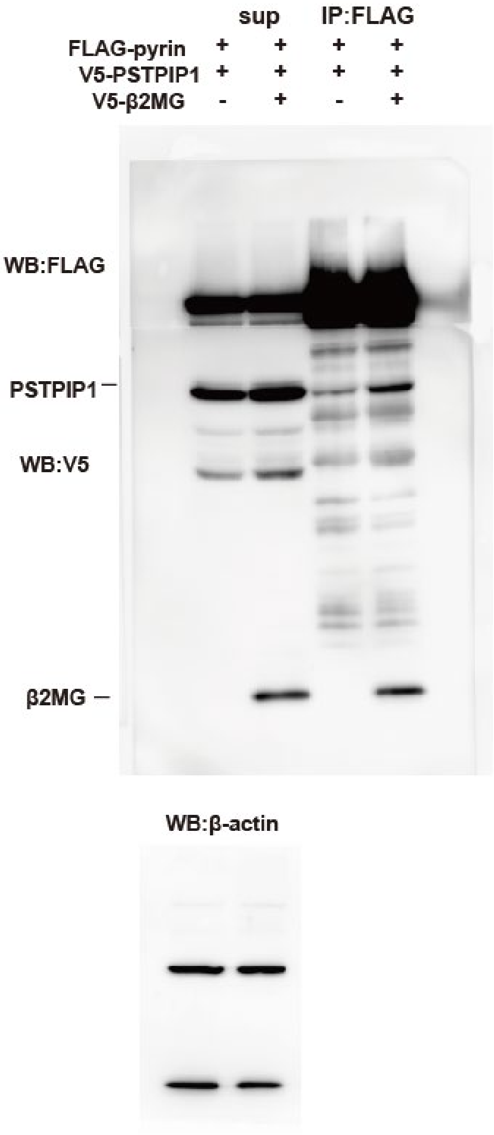

Figure 4F

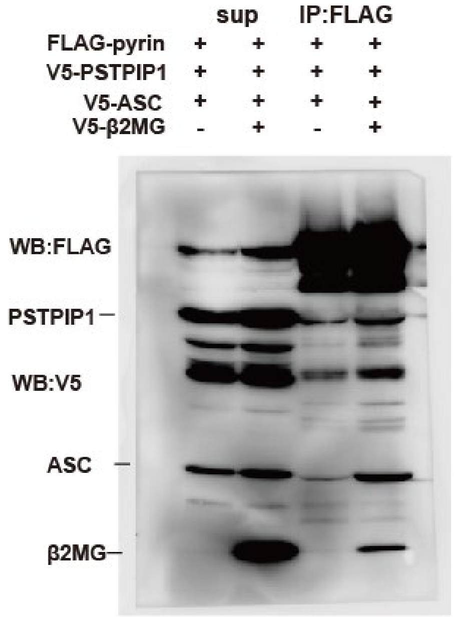

Figure 6A

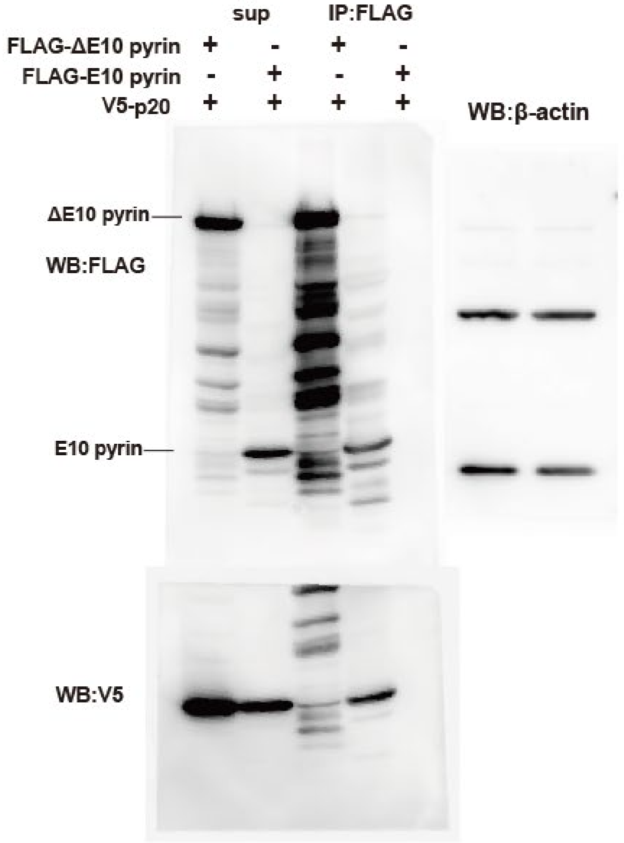

Figure 6C

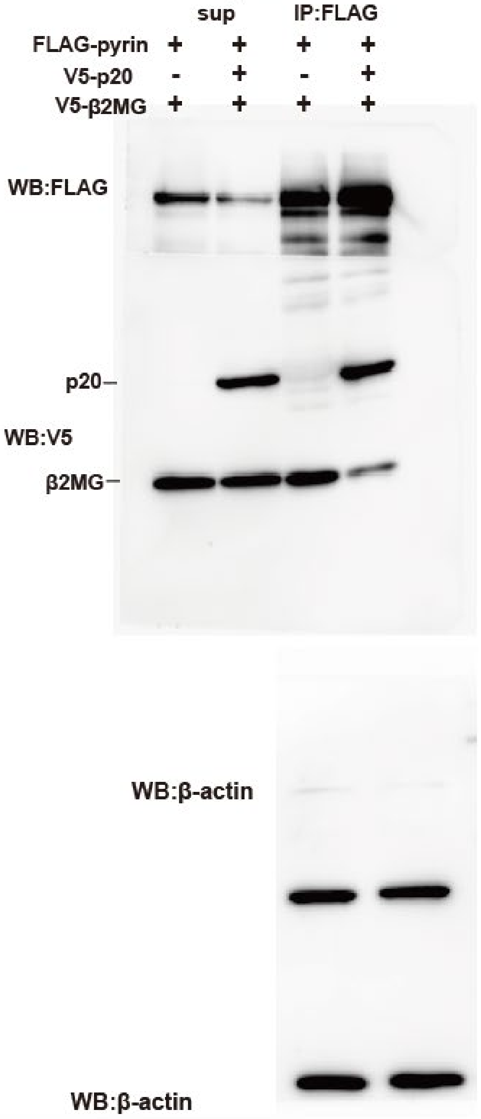

Figure 6B

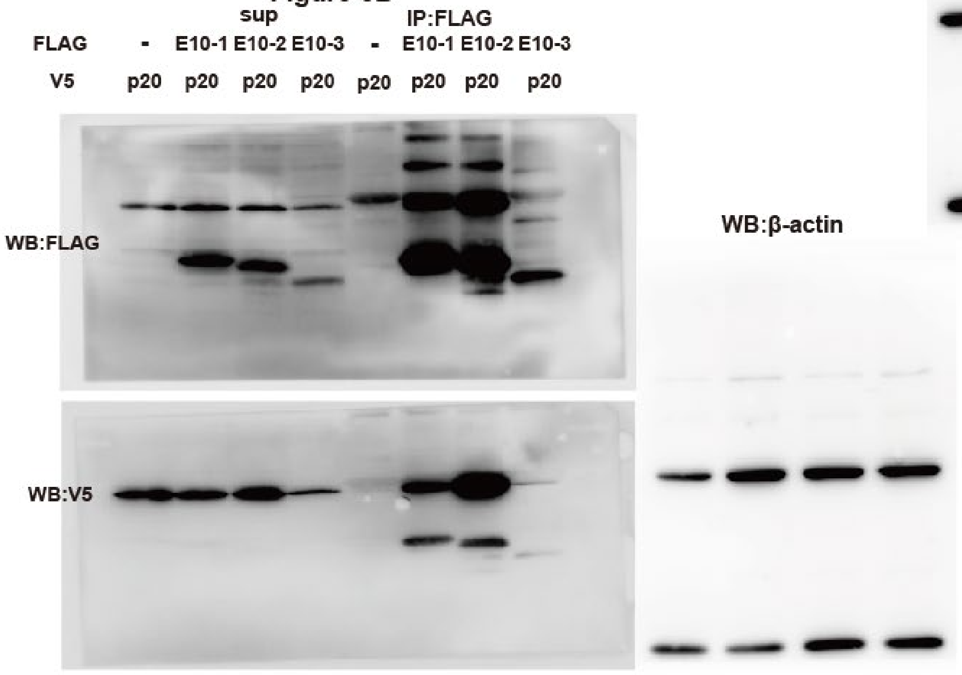

Figure 7A

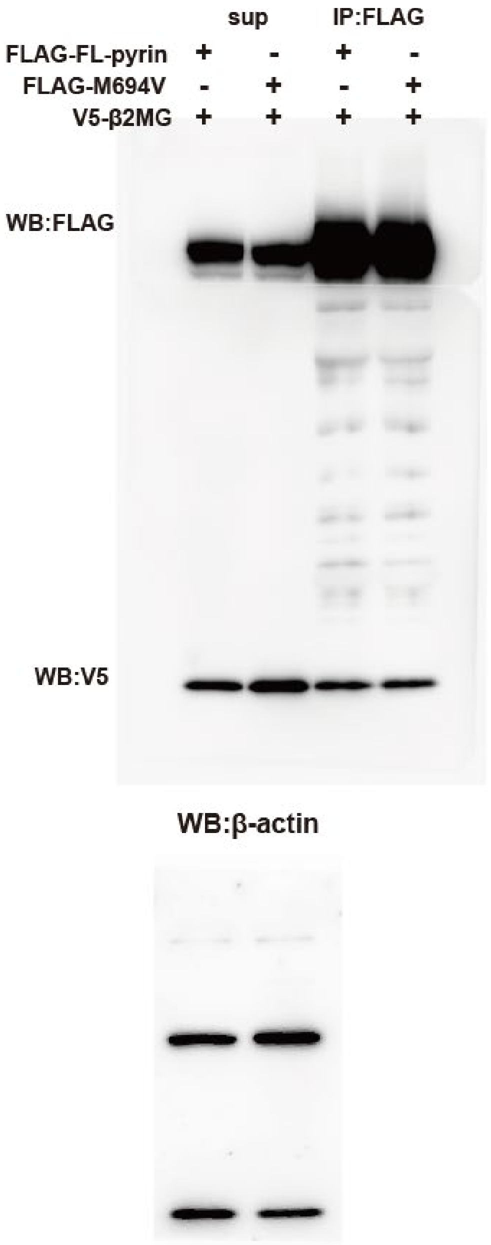

Figure 7B

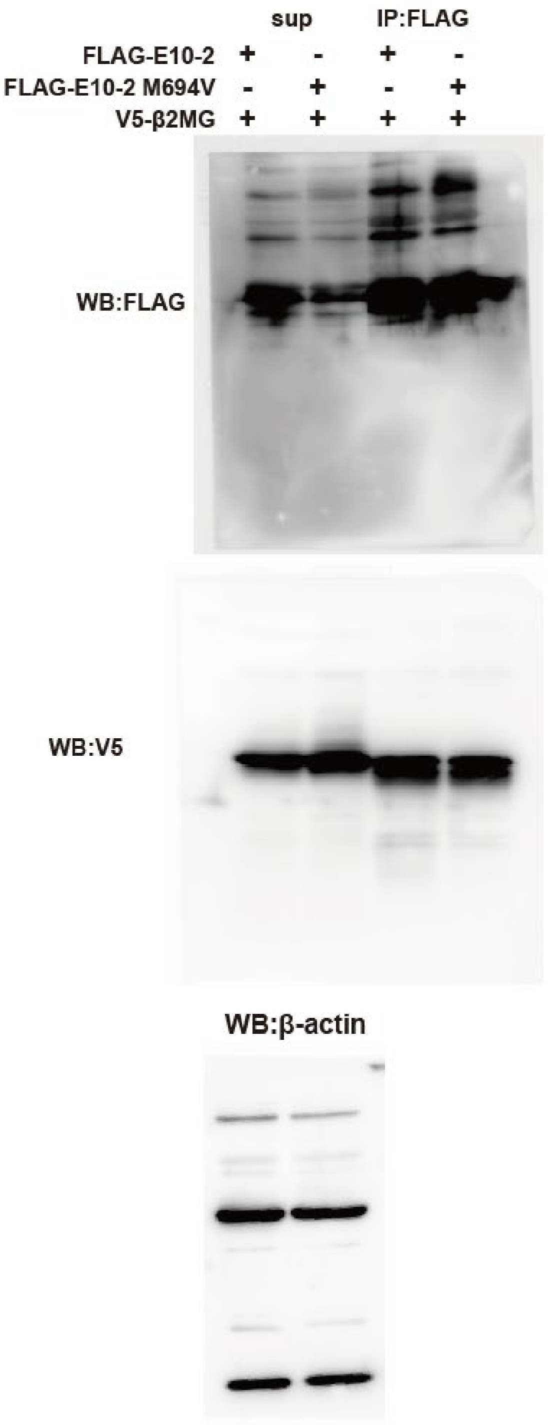

Figure 7C

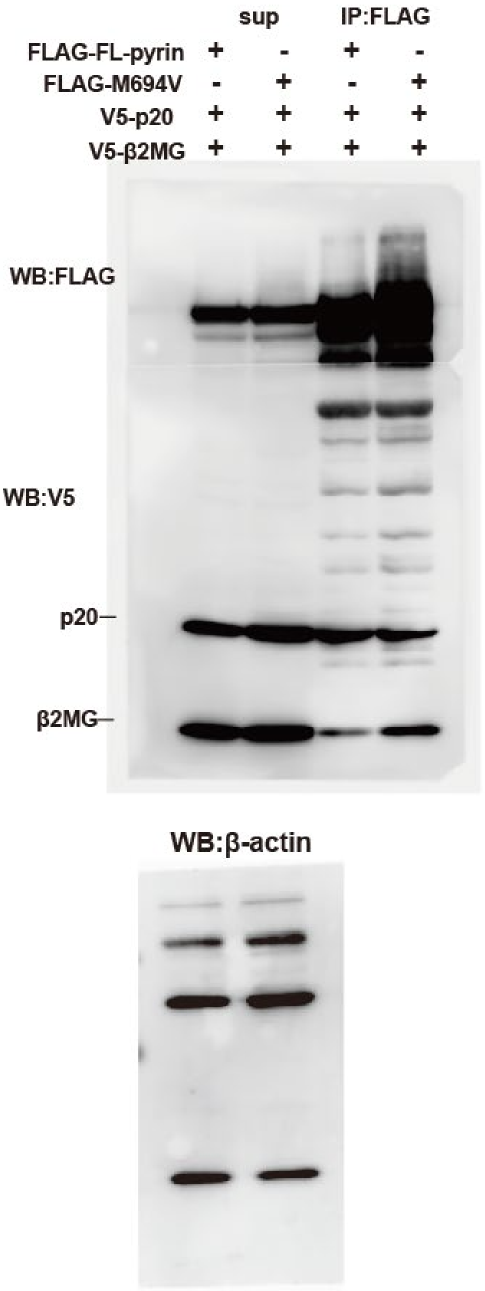

Figure 7D

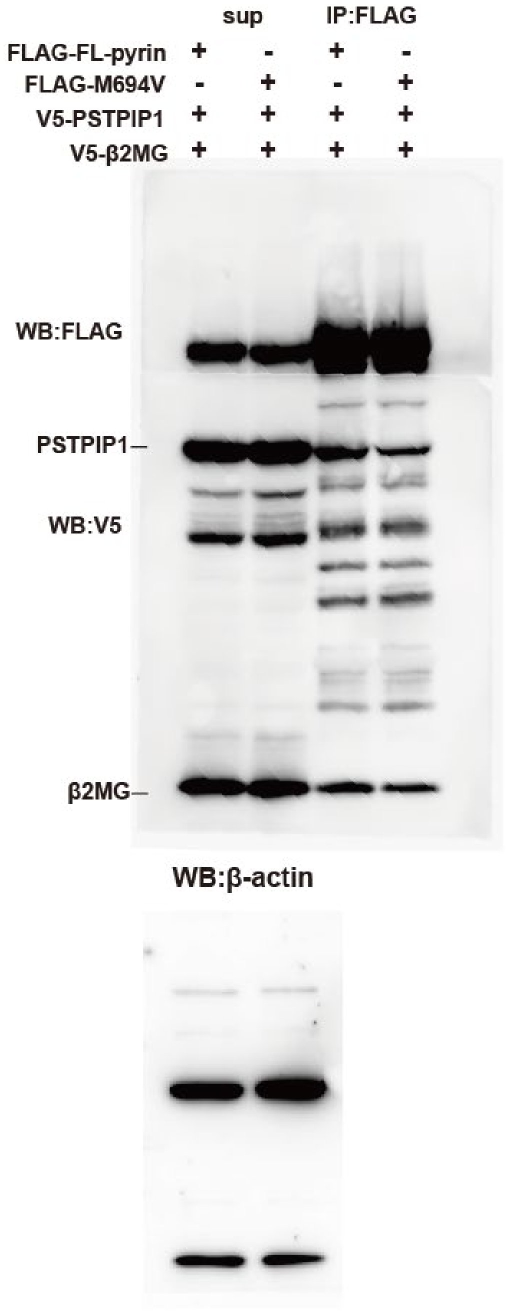

Supplement: Supplementary file 1 — Supplementary Figures. [file 41598_2021_3073_MOESM1_ESM.pdf]
